# Reference values for psychoacoustic tests on Polish school children 7–10 years old

**DOI:** 10.1371/journal.pone.0221689

**Published:** 2019-08-28

**Authors:** Elżbieta A. Włodarczyk, Agata Szkiełkowska, Henryk Skarżyński, Beata Miaśkiewicz, Piotr H. Skarżyński

**Affiliations:** 1 Department of Audiology and Phoniatrics, World Hearing Center of the Institute of Physiology and Pathology of Hearing, Warsaw, Poland; 2 Department of Otorhinolaryngology, World Hearing Center of the Institute of Physiology and Pathology of Hearing, Warsaw, Poland; 3 Department of Teleaudiology and Screening, World Hearing Center of the Institute of Physiology and Pathology of Hearing, Warsaw, Poland; 4 Department of Heart Failure and Cardiac Rehabilitation, 2nd Faculty, Medical University of Warsaw, Warsaw, Poland; 5 Institute of Sensory Organs, Kajetany, Poland; University of Texas at Dallas, UNITED STATES

## Abstract

**Background:**

Good hearing is a fundamental skill that allows children to develop properly, both socially and intellectually. In contrast to defects in inner ear function, however, auditory processing disorders (APDs)–which can affect up to 2–3% of school-children–are not easily identified with basic screening programs and must be diagnosed using special tests. Although such psychoacoustic tests are available, the scores achieved depend highly on the social, cultural, and linguistic characteristics of the population, and norms must be established for each population separately. Reference values are still lacking for the Polish population, especially for children in school-age, so that practitioners must interpret test scores themselves, often intuitively or using potentially biased thresholds from other countries.

**Materials and methods:**

We investigated a sample of 94 Polish schoolchildren with normal hearing, divided into four age groups: from 7 years-olds to 10 years-olds. All children had no speech or language development disorder, learning problem, or symptom of APD. Participants were volunteers who had previously taken part in a large screening study. The group consisted of 56 girls (60%) and 38 boys (40%) with an average age of 8.6 years (SD = 1.1). The test battery included the Duration Pattern Test (DPT), Frequency Pattern Test (FPT), Time-Compressed Speech Test (CST), and Dichotic Digit Test (DDT).

**Results:**

The scores on all tests increased consistently with age. The difference between each age-group for DPT, CST, and left- and right-ear DDT tests was significant (Kruskal–Wallis test, *p*-values = 0.002, 0.006, 0.005, 0.020, respectively), but the effect of age on the FPT test was not (*p*-value = 0.143). The analysis showed a clear and significant separation between a merged group of ages 7 and 8 and another of ages 9 and 10. We, therefore, propose, for each test, separate reference values for these two particular age-groups. Using thresholds based on a 10% quantile, we offer the following reference values for ages 7–8 and 9–10 respectively: DPT, 28.5% and 53.8%; FPT, 18.5% and 27.5%; CST, 68.6% and 77.2%; left-ear DDT, 34.3% and 52.5%; right-ear DDT, 56% and 72.5%.

**Conclusion:**

The scores on psychoacoustic tests to diagnose APD differ between cultures and linguistic backgrounds. Clinicians should, therefore, use norms that have been designed for the population most similar to their patients. Here, we report the use of a test battery designed for the Polish language that accounts for various aspects of APD when screening school children. Together with a full methodology of those tests, we provide norms that can be used as cut-offs in clinical diagnosis. Practitioners are invited to use them to obtain more accurate, evidence-based decisions.

## Introduction

A large group of patients in audiology and phoniatric clinics are children who, despite good peripheral hearing as assessed by objective tests, manifest disorders of speech and language development, articulation disorders, specific difficulties in learning, or another behaviour which suggests hearing loss. These cases are usually connected with disturbed processing of acoustic stimuli at higher levels of the auditory pathway and are known as auditory processing disorders (APDs). It is estimated that as many as 2–3% of schoolchildren [1] can be affected, which in Poland is estimated at 50–60,000 children. Undetected and untreated, APD adversely affects the child’s quality of life [2].

The nature of these deficits makes them very difficult to observe by parents until a child starts regular school and the first symptoms of deficiencies of hearing ability show up in comparison to the child’s peers. Even then, although specially designed tools for identification of APD are available, its diagnosis is challenging, especially in Poland where no established norms exist. As a consequence, Polish professionals in audiology or phoniatric clinics around the country use different, unsystematic approaches to diagnose APD, which results in many false-positives or, less frequently, false-negatives.

As early as 1996, the American Speech-Language-Hearing Association (ASHA) defined and characterized central auditory processes as those processes and mechanisms in the auditory system responsible for the following behavioral phenomena: localization and lateralization of sound sources, sound discrimination (including speech), sound pattern recognition (the ability to compare current sounds with those in long-term memory), time analysis of auditory signals, ability to comprehend distorted speech, and the ability to understand speech in the presence of competing background noise and multi-talker babble [3]. Auditory processing disorders (APDs) are deficits in one of the above hearing functions. In a report from 2005, ASHA pointed out the need to assess the extent of APD in the central nervous system and to evaluate their depth [4]. Electrophysiological tests, such as the auditory cognitive potential P300 and the mismatch negativity, as well as psychoacoustic tests (also called APD tests or central auditory function tests), are used in the assessment. Somewhat later, guidelines from the American Academy of Audiology in 2010 focused on treatment and training regimes to help patients with APD. However, all researchers have emphasised the need for a systematic approach to diagnosing APD at an as early stage as possible [5].

The problem we currently have is that although many of the tests have been created and used for a long time, they still lack explicit norms and clear clinical guidelines. Jerger and Musiek created a list of recommended tests for examining children with APD as well as other tests that could be used for APD screening [1]. However, these authors encouraged every scientist to create their own control groups since, as they argued, the main difficulty in creating universal norms lies in the cultural, educational, and linguistic differences between each population. The test outcome may be affected by both the linguistic characteristics of a particular language as well as by the linguistic experience of the person being tested. Ideally, therefore, there should be normative data for each population, at least at the country level. Similar recommendations for developing individual norms for APD tests are given by Dillon et al. [6]. On the other hand, it should be noted that Katz et al. have presented an opposing view [7], arguing that developing individual norms carries many risks to the quality of the data obtained. Researchers might end up using different methodologies, quality controls, and procedures so that the comparison of data from various research centres becomes difficult or impossible.

In the case of English-speaking patients, there are many studies and guidelines describing how to use and interpret different screening tests, both for adults (SCAN–A) [8] and children (SCAN–C) [9,10]. Some researchers have also investigated other populations and have tried to define normative datasets for adults, e.g. from Denmark [11,12] and Spain [13]. More extensive exploration of psychoacoustic tests has been performed for children, driven mainly by two factors: i) the performance of central auditory processing is highly dependent on age and develops substantially during the first years of school, and so different age groups will show significant differences in scores; ii) APD should be diagnosed as soon as possible to allow countermeasures to be introduced and thus give all children equal chances for development and learning. In this area, research has included languages such as Dutch [11,12], Turkish [14], or an English dialect from New Zealand [10].

In 2007, Fuente and McPherson pointed out that there were no norms for the Polish-speaking population [15]. Recently, two research projects have been conducted in Poland for adults [16] and children [17]. The latter is especially relevant and is an important effort to systematise and structure APD tests for Polish children. However, it has several methodological issues: i) testing was not performed in an ideal experimental setting and was carried out by multiple people in various places around Poland; ii) the methodology of the statistical analysis was not presented in detail, so there was no indication of the type of statistical tests performed or how multiple comparisons were handled; iii) reference values were determined based on the 1^st^ and 3^rd^ quartiles, which to our best knowledge of the field is not an optimal strategy; iv) the study was published only in Polish; v) the results were connected to a commercial product offered to clinics. Although the work of Senderski was no doubt performed with proper diligence and care, we believe that an independent study in a homogenous environment using consistent procedures, rigorous statistics, and fully transparent data is still needed.

In this study, we aim to describe the most relevant characteristics of a standard set of psychoacoustic tests for Polish children of primary school age. The objective is to establish reference values that can be used as cut-offs in medical practice to identify potential problems and disorders. We examine children in the age range of 7–10 years. We chose such age limits because, firstly, such a population is the most important from a practical perspective. In our clinical experience, we mostly see children of such an age. Secondly, the nation-wide program of hearing screening in Poland covers exactly that age range. Thirdly, we believe that APD disorders should be identified as soon as a child starts school. We have therefore focused on the first four levels of primary school. The test battery we used includes tests that assess performance of different aspects of central auditory processing: DPT (Duration Pattern Test) and FPT (Frequency Pattern Test)–the ability to analyze acoustic events over time [18–20], the CST (Time-Compressed Speech Test)–the ability to comprehend distorted speech and understand speech in the presence of noise [21], and the DDT (Dichotic Digit Test)–the ability to separate or integrate disparate auditory stimuli between the ears [1]. Research has shown that these tests are specific and sensitive in detecting a range of central nervous system defects; they are commonly used in diagnosing APD [16,22] and form a part of many widely used test batteries and platforms [22–24].

The purpose of this study is two-fold. Firstly, to provide a systematic, Polish-oriented, and practically proven methodology that could be used to screen APD disorders, based on normative data in a population of Polish children aged 7–10. Secondly, to characterise the collected data and devise appropriate reference values. As a result, both our methodology and presented results permit practitioners to diagnose APD in Polish children systematically.

## Materials and methods

The study was conducted at the Institute of Physiology and Pathology of Hearing in Warsaw, Poland. In total, there were 94 participants aged 7–10 years. All children were recruited from two local public schools after an area-wide hearing screening program (not psychoacoustic) that covered thousands of school-aged children in the Masovian voivodeship. All subjects were volunteers whose hearing was indicated as normal by screening tests: i.e. they had proper articulation, did not have any speech or language development disorders, no learning problems, and did not have any symptoms of APD. The children’s legal guardians were informed of the testing procedures and signed a consent form for their children to participate in the screening program. The form included a statement that the child is aware of the nature of tests to be carried out, and hers/his participation is not again hers/his will. Also, before any psychoacoustic test was conducted oral consents from a representative of the school, the parents and children were obtained. No participant received any form of financial remuneration.

Each child who volunteered was accepted, as long as she or he fulfilled all the following conditions:

1. Peripheral hearing sensitivity within normal limits. Hearing thresholds did not exceed 15 dB HL (pure-tone audiometry using air and bone conduction at 500, 1000, 2000, and 4000 Hz).
2. Absence of CAPD risk factors. Data were obtained from questionnaires which were filled in by both teachers and parents. Questions related to potential articulation disorders, learning problems, impaired development of speech or psychomotor performance, difficulties in understanding speech in noise, memory disorders, or concentration disorder (see examples of questions in the S1 Text).
3. Absence of any indication of ear infections or diseases that occurred within a few weeks preceding the moment of qualification.
4. A participant could not be a student of a music school or systematically participate in dancing or rhythm classes.

Initially, we qualified 100 children, but three of them were not included in the analysis due to their age on the day of the experiment (two children were too old, one was too young), while another three did not complete all tests. Eventually, the group consisted of 56 girls (60%) and 38 boys (40%) and their mean age was 8.6 years (SD = 1.1). The raw data of individual participants is available in S1 Dataset.

### Ethics statement

The authors did not seek specific permission of the Bioethics Committee to conduct the study due to the following reasons. i) The conducted tests have an established protocol, are non-invasive, and routinely used in audiological practice. ii) Participants were primarily those who took part in a large screening program conducted at dozens of schools in the Masovian voivodeship. iii) Since 2008, based on a written agreement, the Institute of Physiology and Pathology of Hearing in Warsaw has implemented a hearing screening program for schools in Warsaw and Masovia in collaboration with the Health Policy Office of the Capital City of Warsaw, the Masovian Local Education Authority, and with supervision of the Committee of Clinical Sciences of the Polish Academy of Sciences. Our study is a side project within the general program, which is set up as a basis for expanding screening procedures.

To ensure compliance with ethics, the authors had access only to information about the gender and age of a child, and no other identifying information. In addition, the research was conducted by a trained person with extensive experience in performing psychoacoustic tests in children, and under appropriate acoustic conditions. Prior to hearing testing, the children’s parents were informed of the testing procedures and signed a consent form for their child to participate in a hearing screening examination. The parents of the children and a representative of the school in which the study was conducted gave oral consent for children’s participation in further studies associated with the psychoacoustic tests. All procedures were conducted in accordance with the Declaration of Helsinki.

### Testing procedures

Each test was performed by an expert with extensive experience in performing psychoacoustic tests in children. All sounds were presented at comfortable listening levels for the subjects, usually 60 dB HL, via headphones (Sennheiser HDA 200) connected to a laptop (HP Compaq nx7400) via a Creative SB1100 sound card. The study was performed either in soundproof booths or sound-treated rooms at the Institute of Physiology and Pathology of Hearing in Warsaw, Poland. All tests were administered in the same order for all participants: firstly FPT, then DPT, DDT, and finally CST. There was a short break between tests, which length was decided by the researcher according to his assessment of the child’s fatigue, but no less than 5 minutes. In total, the whole procedure lasted from 1.5 to 2 hours per person. Before each test, it was confirmed that subjects are willing to participate, and initial instructions were given, together with a few practice examples. The test was started only if it was certain that the participant understood the task. The following psychoacoustic tests were conducted.

#### Duration Pattern Test (DPT)

The original version of DPT [19], implemented as a stand-alone application and available on CD [18], was used. All sounds were presented at a volume of 60 dB HL for both ears. The task was to listen to three 1000 Hz tones, presented bilaterally in a random sequence, and identify the length of each tone (short or long). For example, if the child heard a long tone, a short tone, and a short tone, the correct answer would be long-short-short. The short tone had a length of 200 ms, while the long tone lasted 500 ms. Tones were separated by 200 ms. For each participant, this task was repeated 30 times using randomly generated sequences. The score of the test was the percentage of correctly identified sequences.

#### Frequency Pattern Test (FPT)

The original version of FPT [20], implemented as a stand-alone application and available on CD, was used [18]. All sounds were presented at 60 dB HL for both ears. The task was to listen to three 180 ms tones, presented bilaterally in a random sequence, and identify the frequency of each tone (low or high). For example, if the child heard a high tone, a low tone, and a low tone, the correct answer would be high-low-low. The low-frequency tone was set to 880 Hz, while the high-frequency tone was at 1020 Hz. Between each tone, there was an interval of 200 ms. For each participant, this task was repeated 30 times with randomly generated sequences. The score was the percentage of correctly identified sequences.

#### Time-Compressed Speech Test (CST)

A stand-alone application, available on CD, was used. A Polish version of this test was developed as a result of scientific cooperation between the World Hearing Center of the Institute of Physiology and Pathology of Hearing (Poland) and Brigham Young University Department of Communication Disorders (USA) [25]. All sounds were at 60 dB HL for both ears. The task was to listen to 35 monosyllabic words, presented bilaterally, and repeat them. Each word was compressed in time by a varying percentage–from 25% to 80%. The length of each compressed word was shortened by a specific factor, e.g. a word that usually occupies 1 s and was shortened to 600 ms (i.e. was shortened by 400 ms), so has a compression factor of 40%. The compression factor was increased every five words and included (in a specific order): 25%, 30%, 40%, 50%, …, 80%. The next word was given after the participant repeated the previous one. All words were generated randomly from a pre-defined list. The score of the test was the percentage of correctly repeated words.

#### Dichotic Digit Test (DDT)

A stand-alone application, available on CD, was used. A Polish version of this test was developed as a result of scientific cooperation between the World Hearing Center of the Institute of Physiology and Pathology of Hearing (Poland) and Brigham Young University Department of Communication Disorders (USA) [25]. All sounds were presented at 60 dB HL for both ears. The task was to simultaneously listen to two different digits in the left ear and two different digits in the right ear and then repeat the digits from both ears. The digits were monosyllabic and bisyllabic Polish numbers from 1 through 10 matched for the duration so that the maximum difference in length between the digits presented to the right and left ear did not exceed 230 ms (there are six bisyllabic and four monosyllabic digits in the Polish language). Mono- and bisyllabic digits could be mixed within one trial, e.g. monosyllabic to the left ear and bisyllabic to the right ear. Children were encouraged to guess when they were unsure of a response. There were 60 pairs of digits (30 pairs in the right ear and 30 in the left), generated randomly. The score was the percentage of correctly identified sequences, separately for each ear.

### Statistics

All statistical analyses were carried out in R [26]. For all tests, it was assumed that a *p*-value below 0.05 indicated rejection of the null hypothesis. Mann–Whitney and Kruskal–Wallis non-parametric tests were used to test the differences between two or more groups, respectively. A Benjamini–Hochberg correction was used if multiple comparisons were involved.

All figures were prepared with R’s *ggplot2* package. In the case of box plots, boxes represent the 1^st^ and 3^rd^ quartiles, a horizontal line is a median, and a vertical line shows the range between the lowest and highest value (unless the value exceeded 1^st^ or 3^rd^ quartiles by more than 1.5 of interquartile range). Observations that did not match those assumptions were treated as outliers and were marked with a single point. The density of distributions was estimated with Gaussian kernels (as implemented in the *stat_density* function of the *ggplot2* package).

Reference values were established in specific groups based on the 10% quantile of the distribution of each test. Additionally, in the S1 Text, we show an alternative approach, based on calculating two standard deviations from the mean (as recommended by the American Academy of Audiology).

Comparing our results with other studies required an individual approach because individual data points were not available from the other tests. To get a reliable measure of difference, we divided the procedure into two steps:

1. Approximating the distribution of the data from the other study. In cases where only the mean and standard deviation were available, we assumed that the data followed a Gaussian distribution. Otherwise, if quantiles were available, linear interpolation was used to reproduce the cumulative distribution function.
2. Sampling the data and estimating the difference. We sampled the same number of observations as reported in the original study using either a Gaussian random number generator or the method of inverse transform sampling. Then we calculated the mean difference between the sampled dataset and our data. Lastly, the procedure was repeated 100 times to obtain robust estimates–average difference, together with the standard error. The significance of the result was then established using a Student’s t-test.

## Results

Below we describe the characteristics of the standard test battery of psychoacoustic tests for sample of Polish school-aged children. Collectively, our data allows to devise reference values that can be used to flag children with potential disorders.

We focus on age as the primary determinant of psychoacoustic capability in each test, a parameter which follows consistent findings from various studies around the world [10,14]. Based on the results obtained, it is then possible to calculate reference values for each test that could serve as diagnostic norms. Finally, we compare our data with the results of similar tests reported elsewhere. Other parameters (e.g. gender) we explored were statistically insignificant (as shown in the S1 Text, S5 Table and S1 Fig).

### Age and psychoacoustic test scores

#### DPT test

**Fig 1:**
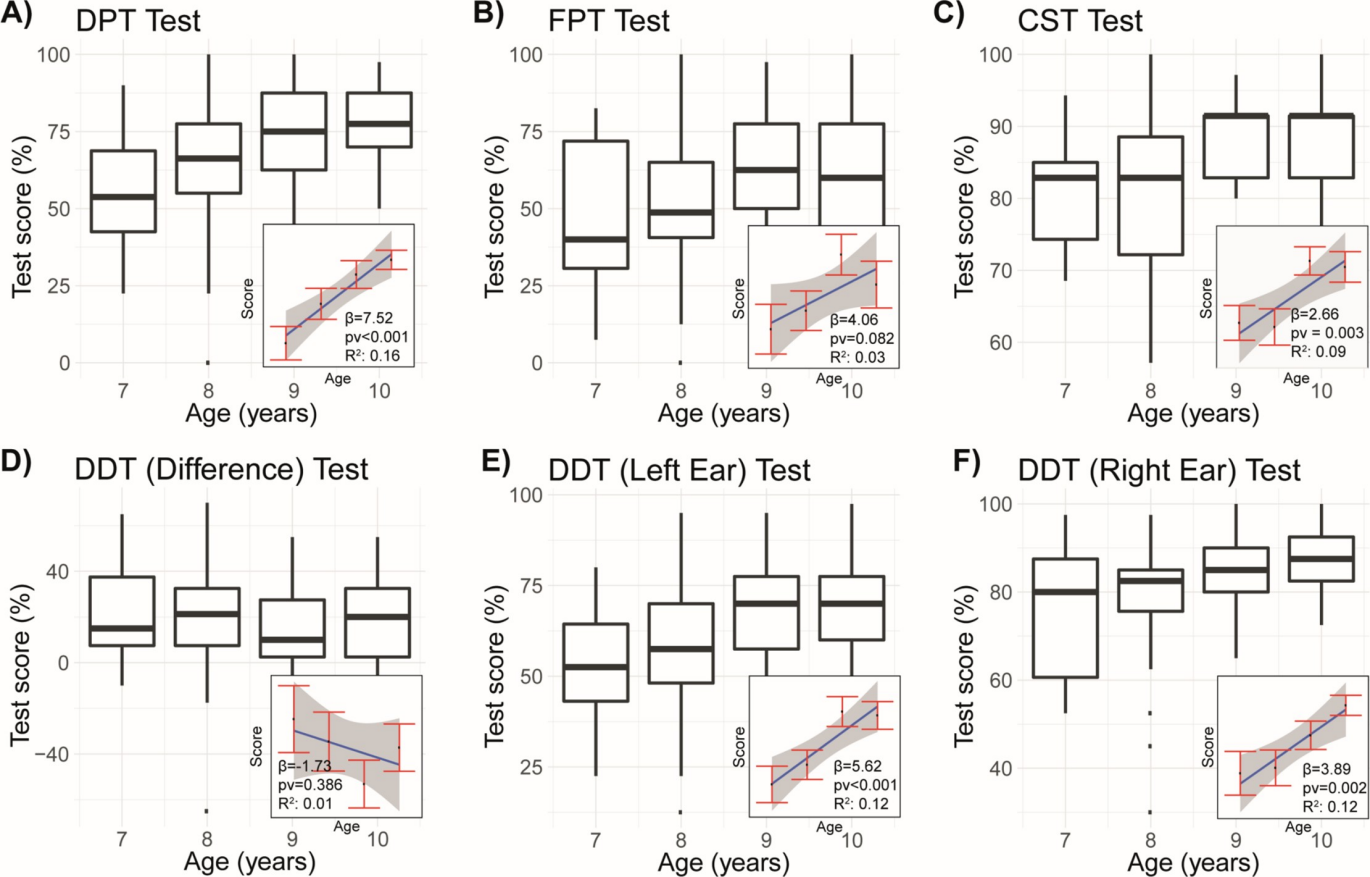
Distributions of test scores show a consistent increase in central auditory capabilities with age. A) DPT Test. B) FPT Test. C) CST Test. D–F) DDT Test separated into scores for left (E) and right (F) ears and the difference in scores between the right and left (D). Distributions are presented as boxplots with single points indicating outliers. In small boxes at the bottom right of each panel are the results of linear regression in which the equation *score* = *α*+*β*∙*age* is fitted. In this way, the effect of age on the score can be seen; also displayed are the magnitude of the effect (*β*), the *p*-value of its significance (*pv*), and the *R*^2^ of the fit.

The mean DPT test score increased from 54% for the group of 7-year-olds up to 77% for the group of 10-year-olds. The standard deviation ranged from 13.3% to 23.6% (Fig 1A). A statistically significant relationship between age and test score was confirmed by a Kruskal–Wallis test (*p*-value = 0.002). Pairwise multiple comparisons of separate age groups (Mann–Whitney test with Benjamini–Hochberg correction) showed a statistically significant difference between the groups of 7-year-olds vs 9-year-olds (median 53.8 vs 75.0, *p*-value = 0.014) and between 7-year-olds and 10-year-olds (median 53.8 vs 77.5, *p-value* = 0.002). Differences in other comparisons did not reach statistical significance (see S1 Table for *p*-values of all comparisons). Basic statistical descriptors of the DPT test scores are shown in Table 1.

**Table 1:** Basic statistics describing the DPT test in each age group.

Age	*N*	Min	Mean (SD)	Median	Max
7	18	22.5	54.2 (19.7)	53.8	90.0
8	30	0.0	65.1 (23.6)	66.3	100.0
9	21	42.5	73.2 (17.7)	75.0	100.0
10	25	50.0	77.3 (13.3)	77.5	97.5
Total	94	0.0	68.1 (20.7)	70.0	100.0

#### FPT test

The mean of the FPT test scores was 45% for 7-year-olds and reached 60% for 9- and 10-year-olds. We present detailed statistics in Table 2. In this case, the relation between age and test score could not be formally proven (Kruskal–Wallis test, *p*-value = 0.143). One reason was the high variability of the test measurements–a standard deviation above 24% for the whole population and above 21% in each group (Fig 1B), which corresponds to almost half the mean in each case. Such high variability does not allow one to reliably confirm the difference between the age groups, despite the tendency previously noted in the means.

**Table 2:** Basic statistics describing the FPT test results in each age group.

Age	*N*	Min	Mean (SD)	Median	Max
7	18	7.5	46.7 (23.7)	40.0	82.5
8	30	0.0	50.8 (24.4)	48.8	100.0
9	21	27.5	63.5 (21.0)	62.5	97.5
10	25	20.0	56.7 (26.2)	60.0	100.0
Total	94	0.0	54.4 (24.4)	50.0	100.0

#### CST test

The mean score of the CST test for 7-year-olds and 8-year-olds was 81% and 80.5% respectively, while for 9-year-olds and 10-year-olds it reached 87.8% and 87.1% respectively. At the same time, the variability of the scores was not high–the standard deviation varied between 7.1% and 10.9% (Fig 1C). A Kruskal–Wallis test confirmed the significance of age as a predictor of CST score (*p*-value = 0.006). Subgroup analysis showed that the difference was substantial, especially between the 7-year-olds or 8-year-olds compared with either the 9-year-olds or 10-year-olds. Specifically, statistical significance was proven with Mann–Whitney test with Benjamini–Hochberg correction for comparison between 7 vs. 9 (median 82.9 vs. 91.4, *p*-value = 0.023), 7 vs. 10 (median 82.9 vs. 91.4, *p*-value = 0.038), 8 vs. 9 (median 82.9 vs. 91.4, *p*-value = 0.023), 8 vs. 10 (median 82.9 vs. 91.4, *p*-value = 0.023). On the other hand, the groups of 7-year-olds and 8-year-olds could not be distinguished statistically (median 82.9 vs 82.9, *p*-value = 0.925), and the same outcome occurred with groups of 9-year-olds and 10-year-olds (median 91.4 vs 91.4, *p*-value = 0.925). We present basic statistical descriptors of CST scores in Table 3.

**Table 3:** Basic statistics describing the CST test results in each age group.

Age	*N*	Min	Mean (SD)	Median	Max
7	18	68.6	81.0 (8.1)	82.9	94.3
8	30	57.2	80.5 (10.9)	82.9	100.0
9	21	68.6	87.8 (7.1)	91.4	97.2
10	25	68.6	87.1 (8.4)	91.4	100.0
Total	94	57.2	84.0 (9.5)	84.3	100.0

#### DDT test

Mean scores in the DDT test for the left ear varied from 53.1% (7-year-olds) to 68.9% (9-year-olds), while the standard deviation varied from 15% to 17.5% (Fig 1E and 1F). By way of contrast, the scores of the DDT test for the right ear were substantially higher, increasing from 76.4% (7-year-olds) up to 87.1% (10-year-olds). Variability of the right-ear DDT was also lower, with standard deviations of 8% to 15.4%. Other statistics are shown in Table 4. For both left- and right-ear tests, we identified a significant statistical relation between age and score on the test (Kruskal–Wallis test, *p*-value = 0.005 and 0.020, respectively). When we compared subgroups between each other for left-ear DDT with Mann–Whitney test with Benjamini–Hochberg correction, differences could be formally confirmed when comparing 7-year-olds with 9-year-olds (median 52.5 vs 70.0, *p*-value = 0.027), and comparing 7-year-olds with 10-year-olds (median 52.5 vs 70.0, *p*-value = 0.027). Similarly, we obtained statistically significant results when comparing 8-year-olds versus 9-year-olds (median 57.5 vs. 70.0, *p*-value = 0.035) and 8-year-olds versus 10-year-olds (median 57.5 vs. 70.0, *p*-value = 0.035). In contrast, groups of 7- and 8-year-olds were statistically indistinguishable (median 52.5 vs. 57.5, *p*-value = 0.479), and the same result occurred for groups of 9- and 10-year-olds (median 70.0 vs. 70.0, *p*-value = 0.886). Subgroup analysis of the right-ear DDT test results showed significance only between 8-year-olds and 10-year-olds (median 82.5 vs 87.5, *p*-value = 0.042), while the other groups did not reach the 0.05 threshold (see S1 Table for *p*-values of all comparisons).

**Table 4:** Basic statistics describing the DDT test results for the left ear (top) and right ear (bottom) in each age group.

**DDT left ear**					
**Age**	***N***	**Min**	**Mean (SD)**	**Median**	**Max**
7	18	22.5	53.1 (16.9)	52.5	80.0
8	30	12.5	57.3 (17.5)	57.5	95.0
9	21	35.0	68.9 (15.0)	70.0	95.0
10	25	37.5	68.1 (15.1)	70.0	97.5
Total	94	12.5	62.0 (17.3)	62.5	97.5
**DDT right ear**					
**Age**	***N***	**Min**	**Mean (SD)**	**Median**	**Max**
7	18	52.5	76.4 (14.6)	80.0	97.5
8	30	30.0	77.3 (15.4)	82.5	97.5
9	21	60.0	82.4 (10.3)	85.0	100.0
10	25	72.5	87.1 (8.0)	87.5	100.0
Total	94	30.0	80.9 (13.1)	83.8	100.0

Most researchers report that patients usually identify speech-related stimuli to their right ear with greater accuracy in comparison to their left ear, a phenomenon known as the right-ear advantage (REA) [27,28]. Many audiologists consider it to be an indication of left-hemisphere dominance for language. In our case, it is a relevant factor in the DDT Test. We investigated the REA of the DDT test score (Fig 1D) by subtracting the score of the left-ear DDT from the score of the right-ear DDT. As reported in most other studies, the right-ear was consistently positive with a mean of around 20%. Although this effect seems to decrease with age, it cannot be established statistically (*p*-value = 0.389). The correlation between the REA and the test scores was not significant either (see S1 Text and S2 Fig for details), as some researchers have hypothesised.

#### Regression model

The above exploration of separate psychoacoustic tests demonstrated that, apart from the FPT test, the obtained scores are statistically related to age. Nonetheless, the performed statistical analysis challenge only the existence of the difference between age groups. On the other hand, as indicated by data, literature and intuition, we would like to show directionality between age and the score of psychoacoustic tests–i.e. to justify that the older the patient, the higher the expected score. Therefore, to formally assess this statement, we carried out linear regression with age as the independent variable and test score as the dependent variable (see regression curves in Fig 1). Specifically, for each psychoacoustic test, we fitted an equation

score=α+β∙age,

where *α* is the intercept and *β* is the coefficient representing the effect of age within the obtained score. Excluding the FPT test, all other tests had a significant coefficient (*β*) for the influence of age on score (*p*-values: <0.001 (DPT), 0.003 (CST), <0.001 (left-ear DDT), 0.002 (right-ear DDT)), with *β* varying from 2.66 to 7.52. For example, in the case of the DDT Test, *β* was 7.52, meaning that an increase of age by one year should give an increase of DDT score by 7.52%. On average, FPT scores also increased with age (*β* = 4.06), but the uncertainty is too high to reliably interpret such a result (*p*-value = 0.082).

### Setting reference values

Comparisons of age subgroups performed in the previous section indicate both i) globally significant relation between age and test scores; ii) a mixed significance when two specific age groups are set side by side. Indeed, when results of multiple comparisons are carefully examined (S1 Table), significant differences, in any of psychoacoustic tests, are detectable between groups of 7-years vs 9-years-olds; 7-years vs 10-years-olds; 8-years vs 9-years-olds; or 8-years vs 10-years-olds. One the other hand, in every psychoacoustic test, no difference could be proven to be statistically justified between the groups of 7-years vs 8-years-olds and 9-years vs 10-years-olds. Together, those findings suggest a visible clustering of 7- with 8-years-olds and 9- with 10-years-olds with respect to scores obtained in psychoacoustic tests. Therefore, we decided to create two larger subgroups for setting the reference values: group I of 7- and 8-year-olds (48 observations), and group II of 9- and 10-year-olds (46 observations). It allowed us to provide reliable and robust statistical estimates.

Specifically, all group I vs. group II comparisons proved to be statistically significant by computing with the Mann–Whitney non-parametric test: DPT (median 60.0 vs 75.0, *p*-value <0.001), FPT (median 45.0 vs. 61.3, *p*-value = 0.036), CST (median 82.9 vs. 91.5, *p*-value <0.001), DDT left ear (median 57.5 vs. 70.0, *p*-value < 0.001), DDT right ear (median 82.5 vs 87.5, *p*-value <0.003).

**Fig 2:**
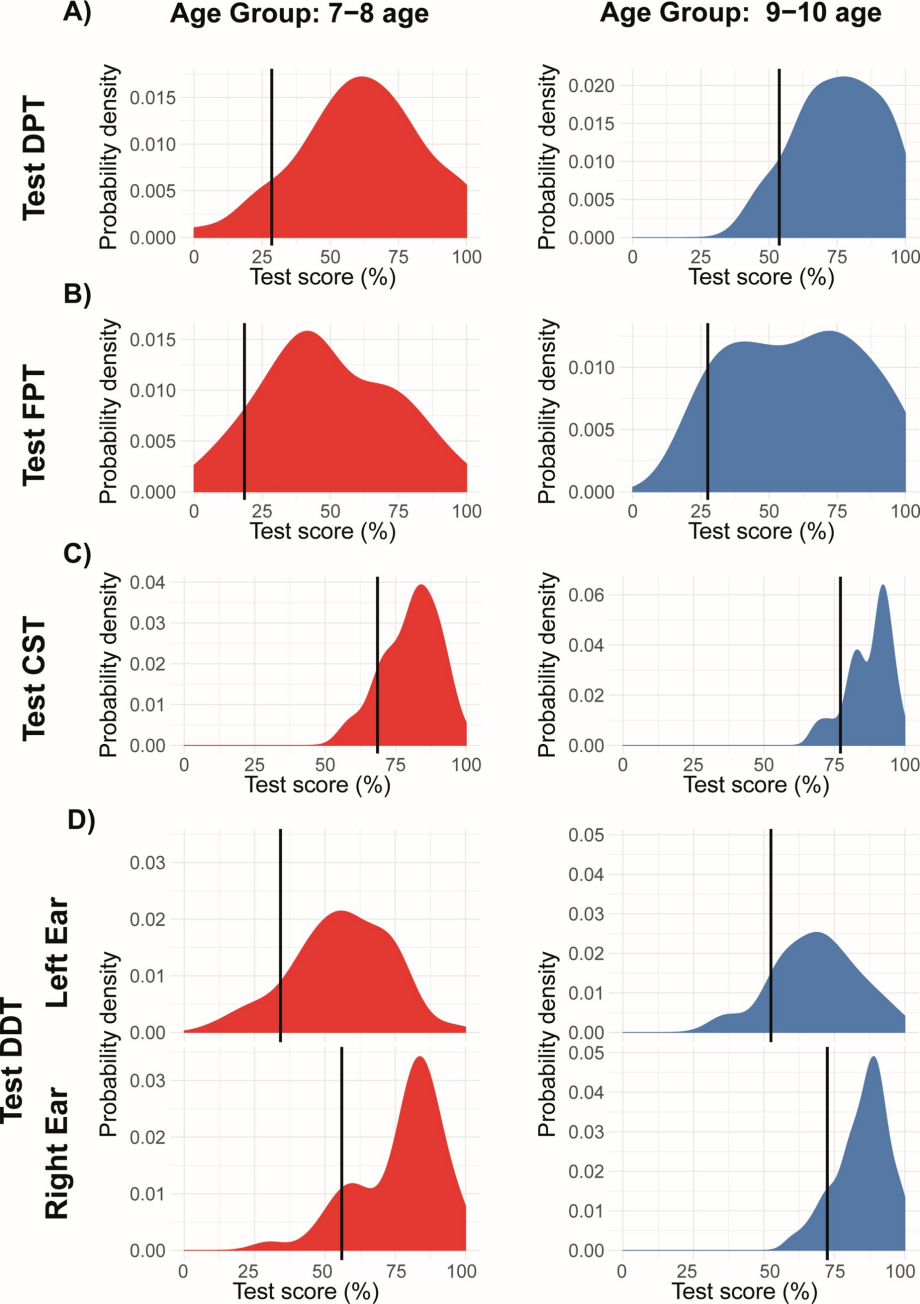
Reference values. Distributions and proposed reference values (solid vertical lines) are given for each test and for two age groups: 7–8 (left, red) and 9–10 (right, blue). A) DPT Test. B) FPT Test. C) CST Test. D) DDT Test. Distributions are shown as density plots (kernel density estimator), with reference values (10% quantile) marked. Compare with Table 5.

In order to choose the optimal method of calculating reference values, we first investigated the type of distribution that underlies the data. By analysing the normality of the observed test scores (see S2 Table), we identified that in the case of CST, FPT, and left-ear DDT, at least two of the age subgroups failed to pass the Cramer–von Mises test (*p*-values >0.05). Therefore, for consistency of analysis, a non-Gaussian approach was followed. Specifically, in each case, we calculated reference values as a 10%-quantile. The reasons behind such a choice were twofold: i) we needed to maintain sufficient specificity of any detected disturbance, and ii) 10% is a threshold considered adequate in other similar studies [9,11,29]. The proposed reference values are reported in Table 5, while comparison with observed data distributions is shown in Fig 2.

**Table 5:** Reference values for selected psychoacoustic tests.

Test type	Age subgroup	Reference value (%)
**DPT**	7–8	28.5
	9–10	53.8
**FPT**	7–8	18.5
	9–10	27.5
**CST**	7–8	68.6
	9–10	77.2
**DDT Left**	7–8	34.3
	9–10	52.5
**DDT Right**	7–8	56.0
	9–10	72.5

When providing reference values it should be noted that the American Academy of Audiology recommends a separation of two standard deviations [5]; although such an approach is, in our belief, not sufficiently reliable (for example, when the distribution is asymmetric), we do report reference values based on ±2 SD in the S3 Table.

### Comparison with other studies

As discussed earlier, it is widely accepted that the performance of central auditory processing is strongly affected by cultural and linguistic background. We, therefore, paid particular attention to how well our results matched those reported by similar studies from other countries.

Unfortunately, in most studies, the distributions of scores are not fully described. As a result, we had to use different approaches to compare findings, depending on the amount of data presented. If only the means and standard errors (deviations) were reported, we assumed a Gaussian distribution. However, if quantiles were given, we approximated the cumulative distribution function by linear interpolation and reconstructed the distribution using the inverse method. Then, comparison with the other study was performed by re-engineering its data points from the approximated distribution and calculating the average difference with our study and standard error by bootstrap (sampling repeated 100 times). Statistical significance was evaluated using Student’s t-test.

Firstly, there is a definite difference between our scores for a childhood population and those of any study involving adults (over 18 years). We compared our findings to studies from the USA [18], Netherlands [11,12], and Poland [16], all of which have reported DPT, FPT, and DDT outcomes. In most cases, the difference between our observed scores and scores for the adult population was substantial and ranged from 12% to 39% for the DPT test; from 28% to 40% for the FPT test; and from 15% to 30% for the left-ear DDT. The only exception was the right-ear DDT, for which scores of two studies from the Netherlands [11,12] were comparable. However, it was not comparable to the study involving American population [18], where the right-ear score was larger by 15%.

**Fig 3:**
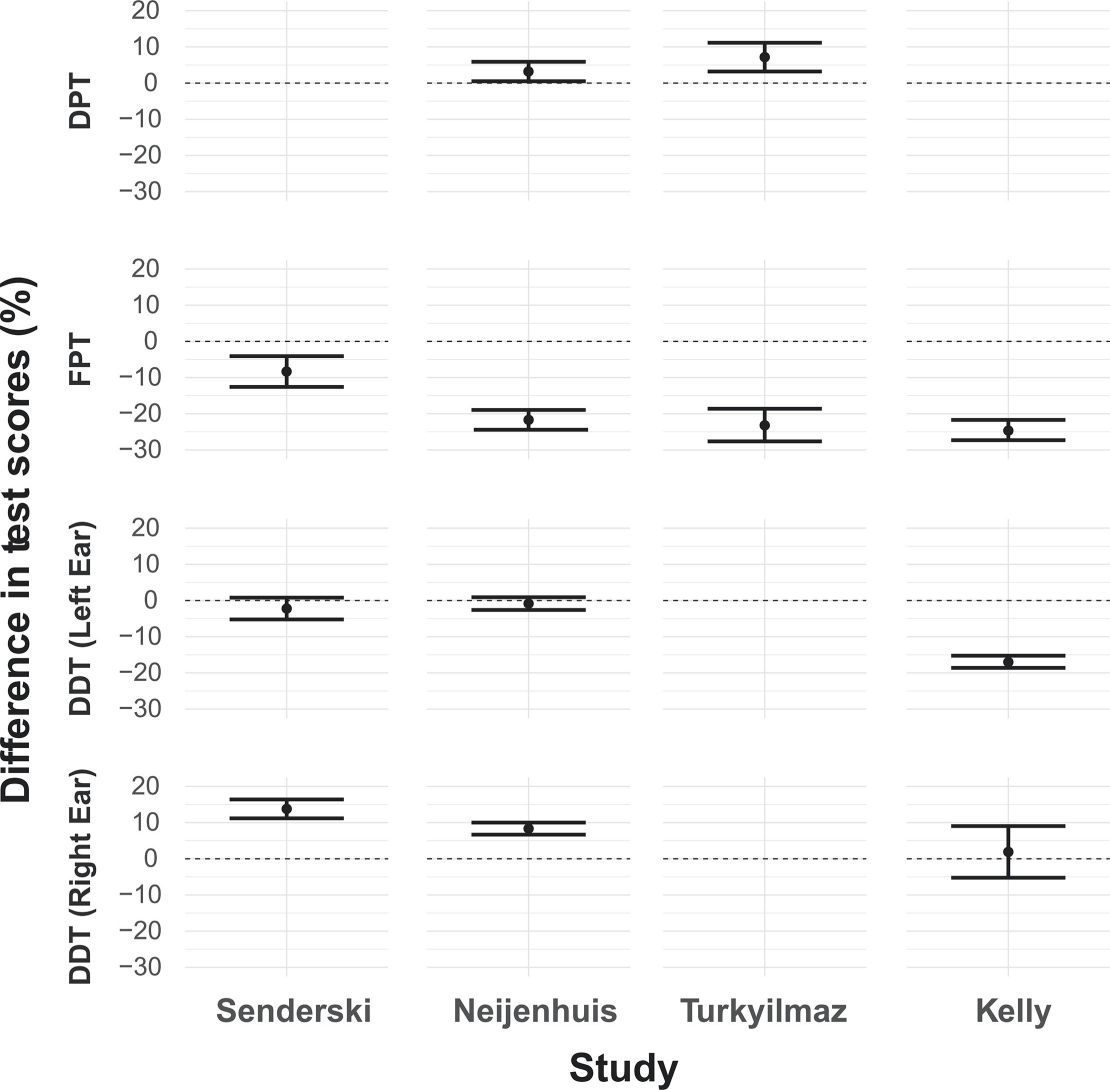
Comparison with other studies. Comparison of results from our study with similar investigations of central auditory processing disorders in children by other authors (left to right: Senderski [17], Neijenhuis [11,12], Turkyilmaz [14], and Kelly [10]). In every case, we show the difference between our findings (dots and error bars) and data published in the corresponding publications (dashed lines at zero). Dots indicate mean difference, while error bars are standard errors. We calculated differences only between comparable groups of observations and then aggregated them by calculating an average difference. Blanks imply no comparable data.

Our main interest lies in comparing our data with other studies involving school-age children. We divide the work into two parts i) a comparison with one study from Poland by [17]; and ii) comparison with studies from The Netherlands [11,12], Turkey [14], and New Zealand [10]. We summarise all comparisons in Fig 3, which shows all the differences between the studies mentioned above and our data.

When the DPT test is considered, our measurements are consistently higher than reported in the literature. Specifically, in comparison with Turkyilmaz et al. (2012) [14] the mean difference is 7.18% (SE = 3.97, *p*-value = 0.03), while in comparison with Neijenhuis et al. (2002) [11] it is 2.98% (SE = 2.37, *p*-value = 0.18).

On the other hand, scores for our FPT test are lower compared with all the others. Differences with the results of Senderski et al. (2016) [17] is –9.39% (SE = 4.24, *p*-value = 0.02); with those of Neijenhuis et al. (2001, 2002) [11,12] it is –21.01% (SE = 2.68, *p-*value <0.01); with Turkyilmaz et al. (2012) [14] it is –23.78% (SE = 4.51, *p*-value = 0.01); and with Kelly (2007) [10] it is –24.51% (SE = 2.80, *p*-value <0.01).

The outcomes of the DDT test vary between left- and right-ear measurements. In the former case, we found an insignificant difference in comparison both with Senderski et al. (2016) [17] (diff. = –2.53%, SE = 3.09, *p*-value = 0.46) and with Neijenhuis et al. (2002) [11] (diff. = –0.55%, SE = 1.91, *p*-value = 0.78). Surprisingly, it is significantly much lower than found by Kelly (2007) [10] (diff. = –16.93%, SE = 1.68, *p*-value <0.01). However, for the right-ear DDT, our population shows a somewhat larger effect than in comparable studies: the difference with Senderski et al. (2016) [17] data is 13.27% (SE = 2.01, *p*-value <0.01), with Neijenhuis et al. (2002) [11] data it is 8.77% (SE = 2.73, *p-*value = 0.03), and with Kelly’s (2007) [10] data it is 1.90%, but insignificant (SE = 7.14, *p*-value = 0.81). However, it should be noted that due to the specifics of the DDT test and differences between languages, the procedure for this test is not uniform between studies. In Polish, there are six bisyllabic and four monosyllabic digits between 1 and 10, and in the test, we administered, it is likely that both types of digits were presented together in a single trial. However, in the English version of this test [30,31] only monosyllabic digits are used (nine digits in total, the number 7 is omitted). Also, in Dutch, only one-syllable numbers are used [11]. As a result, it is reasonable to conclude that the Polish version of this test has a different degree of difficulty than its counterparts in other languages. On the other hand, limiting oneself to only one-syllable digits in Polish would introduce, in our opinion, an even bigger discrepancy with the original test, given the fact that there are only four such digits.

We did not find any study reporting CST test results that would be relevant for comparison with our population.

## Discussion

Diagnosis of central hearing disorders, especially in children of early primary school-age, is currently a major challenge for audiologists and pediatric otorhinolaryngologists. Diagnosis of children is complicated by the co-occurrence of APD with other disorders such as attention deficit disorder, dyslexia, and specific disorders of speech and language development [5,32]. Another difficulty is the lack of clearly defined and widely accepted diagnostic criteria for central hearing disorders. Currently, auditory processing can be assessed using electrophysiological tests such as the late latency response auditory evoked potential, the cognitive potentials P300, and mismatch negativity, as well as behavioural (psychoacoustic) tests. Cortical auditory evoked potentials and cognitive potentials make it possible to assess the functioning of the central hearing system in response to acoustic stimuli. However, behavioural tests provide information on higher hearing functions such as sound localisation and lateralisation, sound differentiation in terms of frequency and duration, and recognition of sound patterns. Research has determined that behavioural tests can provide a reliable diagnosis in children as young as 6 [33].

The early diagnosis of APD is essential, as it can account for as many as 2–3% of school children. What is more, our unpublished studies indicate that in groups with additional risks, APD can happen in 20–30% of subjects with dyslexia and up to 70–80% of cases with specific language impairment. The earlier the child is sent to an audiologist for APD screening, the faster she or he can get help and avoid preventable problems in learning and socialisation [5].

Despite ASHA's consensus on the diagnosis of APD, the set of tests used by researchers in different countries differs significantly due to a lack of developed norms and the increasing number of tests used for screening. All this entails a risk of falsely interpreting a test result as an auditory perception disorder. It underlines the necessity of developing proper diagnostic rules and norms that account for the linguistic and cultural aspects of a population which can then be used in clinical practice. Here, driven by our experience and needs of day-to-day work with children, we recruited a group of 94 children that represented a normative population of Polish children 7–10 years old. They were recruited on a voluntary basis from two public schools from the biggest Polish voivodeship situated in the central part of the country. The schools were both located in a small or medium-sized city and close to the Polish capital, Warsaw. Not only were those two schools relatively large in terms of the number of students (more than 1000 each), but they also have a mix of different socioeconomic and demographic backgrounds. The children’s parents were either Warsaw-based white-collar workers or engaged in rural pursuits. The region is an economically attractive destination for Poles migrating from other parts of the country. Since there were no financial benefits for participation in the study, socioeconomic bias was avoided. We believe that all the measures we took, including choice of location and the number of observations collected, contributed to the representativeness of our sample for the Polish population

The design of the study allowed us to determine norms for four tests: DPT, FPT, CST, and DDT (both ears). Each test measures different aspects of central auditory processing. Despite a multitude of available tests, we believe such a battery is an appropriate setting for screening a general population of children. It contains both non-verbal tests (DPT, FPT) as well as those that use speech stimuli (words in CST; digits in DDT). Furthermore, all of these tests have a well-established place and significance in the literature [8,9,18] and are suitable for children [34]. What is more, Polish versions of CST and DDT were designed under the supervision and training of Frank Musiek from Brigham Young University Department of Communication Disorders. The software that facilitates the use of this test battery is available upon contact from our clinic (i.e. the Institute of Physiology and Pathology of Hearing in Warsaw). We do not assert that our choice of tests for APD screening is the best possible, as this is probably a matter of philosophical discussion, still ongoing [35], and additional factors, both culture- and language-specific, must be taken into account. Nonetheless, based on our long experience, we are convinced that our proposed tests are good enough to screen children for problems with APD in Poland, and taking into account the findings presented here, reliable and practical enough to administer by an audiologist in their day-to-day work.

Specifically, we have proposed reference values of all tests for two groups: 7–8 year-olds and 9–10-year-olds. The reference values can be used as thresholds in diagnosing APD among the general child population of Poland. Linear regression showed a clear tendency for test scores to increase with age. However, we could not define reliable cut-offs for each age separately, because of the lack of significance of some of the between-age comparisons. Part of the reason was substantial variability in the data, especially in the case of FPT test where a strong relation to age could not be established. In our opinion, a large variability should be expected from any child-based psychoacoustic test, since central auditory processing is tightly linked to the child’s intellectual progress, which can vary substantially between children of the same age. It might also be suggested that frequency recognition is driven to a substantial extent by unknown factors not correlated with age. Indeed, the environment of one’s early childhood can have a considerable effect on the heterogeneity of auditory abilities, as observed in psychoacoustic test scores. Possibly, a larger sample is needed to make more reliable statistical inferences and set norms for all age strata.

At the same time, in some cases, the distributions of those tests differed significantly from Gaussian, sometimes being skewed with long left tails (see Fig 2). The non-normality of our data was also the main reason behind choosing a quantile-based approach to determine reference values, instead of the standard-deviation approach (as in [11]). We believe that, due to non-symmetric data, the latter approach is too conservative and allows for too many false-negatives in the diagnosis.

It must also be noted that in our data, one outlier is present. Specifically, a child (boy) with identifier id65 (see S1 Dataset) scored 0% in DPT and FPT tests–he did not correctly guess any of the presented sound sequences. In preliminary data analysis, we double-checked that this was not a mistake in data preprocessing or test reporting. Therefore, although it is an outlier in comparison to other data points, it is not an incorrect observation. Moreover, it was not unexpected. In our clinical practice, we do occasionally examine children that obtain a zero score in some of the psychoacoustic tests. Additionally, we checked whether the conclusions of our study would change, if we excluded the mentioned outlier. We observed no qualitative differences with results reported in the primary analysis (see S6 Table and details in S1 Text).

Some discussion is needed concerning our recommended cut-offs in the FPT test. The primary relation between age and the score in this test was found to be non-significant, both by non-parametric group comparison tests and linear regression. At the same time, comparison between larger age groups, i.e. 7–8-years-olds vs 9–10-year-olds, did reach significance. Taking the evidence as a whole, however, one should be cautious when interpreting results of the FPT test. The scores in this test showed high variability within age subgroups (average coefficient of variation 0.45 versus 0.33 for other tests). Consequently, even weak performance in recognising frequency does not necessarily correspond to an abnormal condition and, at least with respect to our normative data, could be expected. Therefore, our reported FPT thresholds are relatively low, which seems sound when the normative data has high variability. In our opinion, the cut-offs provided are well established.

The human auditory system is not symmetric, resulting for most people in a so-called Right Ear Advantage (REA) that usually decreases from childhood to adolescence until the mid-twenties, and then stabilises at a low level (3–5%). Thereafter, it starts to increase with age [36]. The implied reasons for this phenomenon are numerous: purely physiological, evolutionary, or environmental. According to the paradigm, a decrease in REA should be observed in school children. Our data do not contradict this hypothesis since REA decreases at the level of the means. However, it is also not supported either, because the observed tendency is not statistically significant, probably because our study group was too small to detect the REA dependence on age. In order to thoroughly test the REA hypothesis, we would need to use a wider age-span of children over 10 years old. For example, Neijenhuis showed a decrease in REA, but only between 10–12-year-olds, 14–16-year-olds, and adults [11]. For very young children, contradictory findings have been reported, e.g. Kelly reports only a small decrease in REA with age (from a very small initial value) [10], while Moncrieff found an inverted-U dependence between REA and age [37]. Relevant here could also be the specifics of the Polish version of DDT test used to estimate REA, which was discussed earlier in the Results section (see also below).

In recent years, a number of papers have been published describing tests used for both adults and children in different countries [10,11,13,14,16,17]. Of course, it is necessary to analyse children separately from adults, a fact confirmed by the comparison of our results with data reported in studies involving adults from other countries [18] as well as from Poland [16]. When we compared our findings with child-focused studies [10,14,16,17], no clear tendency can be observed. For any of the compared studies, some of the tests showed higher scores, while others were lower or statistically indistinguishable. However, from the perspective of single tests, a slight advantage of our population in the DPT Test and a considerable disadvantage in the FPT Test were seen.

Results of the left-ear DDT Test were usually comparable with other studies, whereas our subjects obtained much higher scores in right-ear DDT. However, as mentioned in the Results section, the specifics of the Polish language in terms of the number of syllables for digit names, and its impact on the design of the Polish version of DDT test made it difficult to compare results with those from DDT tests in other languages. It is not easy to say whether the differences in the number of syllables made the test easier or harder.

Special attention should be given to the work of Senderski and colleagues, in which the authors analysed a population similar to ours. Nevertheless, the results show substantial differences. One reason could be the way the tests were performed: in our study children were assessed by a small group of experts from the Institute of Physiology and Pathology of Hearing in Warsaw using the same equipment and exactly the same procedures. On the other hand, the tests reported by Senderski were collected from various clinical and educational centres around Poland. Discrepancies between our data and the work of Senderski should be resolved in future work, possibly by conducting a more extensive study with a consistent experimental protocol.

Taken together, differences between our data and studies from other countries should be explained by the way different cultural and linguistic backgrounds influence auditory processing abilities, which leads to reported discrepancies in how fast children from different countries develop various aspects of auditory processing [34]. Apart from factors such as the unique characteristics of a given language, the difficulty of completing tests such as CST or DDT depends on cultural, educational, and sociological factors. Especially in the case of the DPT and FPT tests, finding differences with other studies might be questionable, since, at first glance, these are non-speech tests, without a direct relationship to the subject’s language. Nonetheless, both those tests measure the capabilities of higher auditory pathways and reflect a combination of multiple factors. Specifically, populations from different countries are subject to various confounding variables that can affect the results of tests such as FPT and DPT. One of these is the fact that Polish children start education later than most children in developed countries—education is compulsory from the age of 6 in Poland, whereas in Western Europe and the United States formal education typically begins at age 5. Secondly, the specificity of a given language substantially influences the environment, in which all central auditory abilities, not only linguistic, are developed. For example, tonal languages, like Mandarian, in comparison to the non-tonal language, potentially allows their users to be more sensitive to the sound frequency. Those are only a few factors among many that should not be overlooked when interpreting observed results. All of this implies that comparing results of our studies with those from other researchers shows that norms for psychoacoustic tests always need to be determined with respect to i) type of test; ii) age of patients; and iii) the particular population.

Our study provides a methodology of screening children for APD, which is adjusted to Polish linguistic and cultural specifics. Full procedures, software, and assistance in the practical implementation of the test battery are available upon contact with our clinic. The work is rounded off with diagnostic threshold values obtained from the normative dataset.

Several aspects of our study that limit its applicability must be noted. Firstly, sample sizes in some cases were insufficient to unambiguously prove or discard the hypothesis about an apparent trend with age. Because of relatively small numbers, we had to merge groups of 7- and 8-year-olds and 9- and 10-year-olds. An enlarged study might make it possible to determine reference values for each age group reliably. Secondly, participants of our study came from the Masovian voivodeship, which represents 14% of the Polish population. There could be some factor at work that limits generalising from this population to the whole country. Thirdly, our comparison with results from other studies was based on data provided in publications, which were sometimes very sketchy. To perform comparisons, we took a conservative approach, but a direct sample-by-sample comparison would be considerably more accurate.

## Conclusions

1. We collected normative data for Polish children aged 7–10 (level 1–4 of primary school) in terms of performance on psychoacoustic tests related to APD disorders.
2. Reference values used for the diagnosis of APD must be adjusted to both the population involved and the age of the patient.
3. We propose both a systematic test battery and cut-off thresholds that can be used by audiologists to identify APD among Polish school children 7–10 years old.

## Supporting information

S1 Text Supplementary methods and results. (DOCX)

S1 Table Detailed results of subgroup analysis with multiple comparison. (DOCX)

S2 Table Tests for normality to verify distribution of data. (DOCX)

S3 Table Comparison of reference values calculated using different approaches. (DOCX)

S4 Table Distribution of patients’ gender. (DOCX)

S5 Table Exploration of age and gender effect. (DOCX)

S6 Table Influence of the atypical observation on the study results. (DOCX)

S1 Fig Distributions of test scores according to age group and gender. (DOCX)

S2 Fig Correlation between test scores and Right Ear Advantage (REA). (DOCX)

S1 Dataset All individual measurements for DPT, FPT, CST and DDT tests collected in the study. (XLSX)
