# Supplementary material for: Reference values for psychoacoustic tests on Polish school children 7–10 years old
Source: PLoS One. 2019 Aug 28;14(8):e0221689. doi: 10.1371/journal.pone.0221689 (PMC6713444; doi:10.1371/journal.pone.0221689)
Supplement: S1 Text — (DOCX) [file pone.0221689.s001.docx]

**S1 Text. Supplementary methods and results**

of manuscript

Włodarczyk E, et al., Reference values for psychoacoustic tests on Polish school children 7–10 years old

Table of Contents

[Qualification for the study – questionnaire 2](#_Toc15458361)

[Subgroup analysis of age-groups 3](#_Toc15458362)

[Testing normality 4](#_Toc15458363)

[An alternative approach to reference values 6](#_Toc15458364)

[DPT Test 6](#_Toc15458365)

[FPT Test 6](#_Toc15458366)

[CST Test 7](#_Toc15458367)

[DDT Test 7](#_Toc15458368)

[Additional exploration 8](#_Toc15458369)

[Investigation of gender-wise effects 8](#_Toc15458370)

[Investigation of interdependence between right ear advantage and test scores 11](#_Toc15458371)

[Influence of the outlier on the obtained results 13](#_Toc15458372)

[References 14](#_Toc15458373)

# Qualification for the study – questionnaire

Parents and teachers of children that volunteered for the study were asked to complete a questionnaire, in which, among others, the following questions were asked to identify problems and symptoms related to CAPD:

1. Does your child demonstrate any problems relating to hearing?

2. Is your child’s speech development delayed of disturbed?

3. Does your child often ask you to repeat sentences?

4. Does your child have problems with social contacts?

5. Does your child have decreased attention focus?

6. Does your child have dyslexia?

7. Does your child have problems with learning foreign languages?

8. Does your child have frequent otitis?

There were two additional questions with multiple-choice responses:

9. Does your child ask you to repeat questions? Responses: never, seldom, often, very often.

10. What is your child’s school performance? Responses: very poor, poor, good, very good.

# Testing normality

An essential step in characterising our data was to establish a distribution that could be used as a model for test scores. To achieve this, we carried out an extensive analysis of normality by performing four statistical tests: Cramer–von Mises, Anderson–Darling, Lilliefors, and Wilk–Shapiro. Corresponding *p*-values are presented in S2 Table.

Within each test, at least several groups of measurements needed to be treated as non-Gaussian distributions. Therefore, in general analysis, we cannot assume normality of the data as this would distort the final results. As a consequence, we chose:

1. Mann–Whitney tests for two-sample comparisons
2. Quantile-based approach for identifying reference values.

For a comprehensive analysis of the type of distribution underlying our data, we used the following tests: Cramer–von Mises, Anderson–Darling, Shapiro–Wilk, and Lillefors. All were computed based on implementations available in R (base or nortest package).

# An alternative approach to reference values

Driven by the type of the distribution of our data (see the previous section of Supplementary Information), in our primary analysis, we used 10%-quantiles to established reference values to be used as cut-offs in diagnosis. However, recommendations from audiologists’ guidelines propose a method based on calculating two standard deviations from the mean to define norms (Audiology 2010). On the other hand, this inherently assumes that the data follows a Gaussian distribution, while we discovered that the distribution of our measurements is skewed and an assumption of normality of the data would not be valid. In such a case, using standard deviations would distort identification and screening and would result in many false-negatives (too conservative). Such an opinion is also shared by other authors (Keith 2000, Higson, Haggard, and Field (1994), Neijenhuis et al. (2002)). In the main text, we presented reference values based on a 10%-quantile. Here, for comparison, we report in S3 Table an alternative approach that utilises standard deviations.

In each case, the threshold is determined to be much lower by the method of 2SD, sometimes even extremely low (e.g. DPT or FPT Tests). It is likely to lead to many false-negatives (not discovered diseases) and low sensitivity of the diagnosis.

# Additional exploration

For the sake of completeness of our analysis, below, we present further investigations of our datasets in different directions. Specifically, we investigated a) potential differences between male and female children; b) whether the magnitude of right-ear advantage would predict, to some extent, the outcomes in any of the psychoacoustic tests. As none of the above effects proved to be statistically significant, we did not include it within our discussion in the main paper. Nonetheless, they may be of interest to other researchers on this topic.

## Investigation of gender-wise effects

Possibly, some of the variability within observed outcomes could be accounted for by gender-specific factors. Therefore, we checked how test performance differed between girls and boys within our dataset by performing linear regression and comparison of subgroups. In S4 Table distributions with respect to gender are presented.

Firstly, for each psychoacoustic test, we fitted a linear regression model with interactions between age and gender:

$$score=\alpha+\beta_{1}\cdot age+\beta_{2}\cdot gender+\beta_{3}\cdot age\cdot gender,$$

where:

$\alpha,\beta_{i}$ – are the coefficients to be fitted

$age$ – is a variable representing the age of the participants

$gender$ – is a variable representing the sex of the participants, with 0 = female and 1 = male

The results of estimating the above equation using our dataset are summarised in S5 Table. None of the coefficients related to gender was found to be significant (*t*-test *p*-values: 0.13–0.92) with respect to any of the test scores. Therefore, our data does not justify the hypothesis that gender-specific factors influence psychoacoustic skills of children. To visualise this result (S1 Fig), we replicated Fig 1 of the main paper by additionally dividing age subgroups with respect to gender. When groups of females and males were compared with each other (within the same age groups), they could not be distinguished statistically (Mann–Whitney *p*-values 0.09–0.91).

## Investigation of interdependence between right ear advantage and test scores

Within the main paper, we discussed the right ear advantage (REA) that results from a child’s performance in the DDT test. Specifically, our laterality index is defined as:

$$REA=DDT_{right}-DDT_{left}$$

It was hypothesised whether the difference between right-ear and left-ear perception could be used as a general predictor of central auditory processing abilities. Following such an idea, we explored correlations between REA and performance in each test, which are shown in S2 Fig. All test scores (apart from DDT) are negatively correlated with REA (DPT, FPT: –0.18; CST: –0.19, Pearson correlation coefficient), but not statistically significant (test for association *p*-values: 0.07–0.09). The correlation between DDT tests and REA are strong and significant, but this is mainly a result of their definition and inherent dependence, so it cannot be used to justify the initial hypothesis. In consequence, although no specific conclusion regarding the importance of laterality index could be justified, there is a slight indication that the higher the difference between the performance of right and left ears, the weaker is the central auditory processing ability. Nonetheless, either the laterality index based on DDT test is not specific/sensitive enough, or there is an important, unknown co-variate that prevents us from discovering a formal dependence.

# Influence of the outlier on the obtained results

In our data, there was one child (a boy) who scored 0 in DPT and FPT tests – he did not correctly guess any of the presented sequences. On the other hand, he scored 71.45% in CST, 32.5% in DDT (left ear), and 65% in DDT (right ear) – (observation “id. 65” in the Supplementary data). During preliminary work with the data, we double-checked that this was not a mistake in data preprocessing or test reporting. Additionally, we checked what the result of our analysis would be if we excluded this observation.

We concluded that there were no qualitative differences with the results reported in the main paper (see S6 Table). In consequence, for the consistency and transparency of the results, we decided to leave the full dataset that we collected as it was.

# References

Audiology, American Academy of. 2010. “American Academy of Audiology Clinical Practice Guidelines: Diagnosis, Treatment and Management of Children and Adults with Central Auditory Processing Disorder.” [http://www.audiology.org/resources/documentlibrary/Documents/APD Guidelines 8- 2010.pdf](http://www.audiology.org/resources/documentlibrary/Documents/APD%20Guidelines%208-%202010.pdf).

Higson, Josephine M, Mark P Haggard, and Diana L Field. 1994. “Validation of Parameters for Assessing Obscure Auditory Dysfunction – Robustness of Determinants of OAD Status Across Samples and Test Methods.” *British Journal of Audiology* 28(1): 27–39.

Keith, Robert W. 2000. “Development and Standardization of Scan-c Test for Auditory Processing Disorders in Children.” *Journal of the American Academy of Audiology* 11(8):438–45.

Kelly, Andrea. 2007. “Normative Data for Behavioural Tests of Auditory Processing for New Zealand School Children Aged 7 to 12 Years.” *Australian New Zealand J Audiology* 29(1): 60–64. doi:[10.1375/audi.29.1.60](https://doi.org/10.1375/audi.29.1.60).

Neijenhuis, Karin, Ad Snik, Gertrude Priester, Sanne van Kordenoordt, and Paul van den Broek. 2002. “Age Effects and Normative Data on a Dutch Test Battery for Auditory Processing Disorders.” *Int J Audiol* 41(6): 334–46.

R Core Team. 2015. *R: A Language and Environment for Statistical Computing*. Vienna, Austria: R Foundation for Statistical Computing. <https://www.R-project.org/>.
