# Supplementary material for: Reference values for psychoacoustic tests on Polish school children 7–10 years old
Source: PLoS One. 2019 Aug 28;14(8):e0221689. doi: 10.1371/journal.pone.0221689 (PMC6713444; doi:10.1371/journal.pone.0221689)
Supplement: S1 Table — (DOCX) [file pone.0221689.s002.docx]

**S1 Table.** **Detailed results of subgroup analysis of the relation between age and APD tests**. P-values of the Mann–Whitney nonparametric test, examining the differences between age groups of the DPT, with correction for multiple comparisons (Benjamini-Hochberg). The asterisk (*) indicates statistical significance (p<0.05).

| **DPT Test** | | | |
| --- | --- | --- | --- |
|  | Age: 7 | Age: 8 | Age: 9 |
| Age: 8 | 0.11 | NA | NA |
| Age: 9 | 0.01* | 0.31 | NA |
| Age: 10 | 0* | 0.07 | 0.45 |
| **CST Test** | | | |
|  | Age: 7 | Age: 8 | Age: 9 |
| Age: 8 | 0.92 | NA | NA |
| Age: 9 | 0.02* | 0.02* | NA |
| Age: 10 | 0.04* | 0.02* | 0.92 |
| **Left-ear DDT Test** | | | |
|  | Age: 7 | Age: 8 | Age: 9 |
| Age: 8 | 0.48 | NA | NA |
| Age: 9 | 0.03* | 0.03* | NA |
| Age: 10 | 0.03* | 0.03* | 0.89 |
| **Right-ear DDT Test** | | | |
|  | Age: 7 | Age: 8 | Age: 9 |
| Age: 8 | 1 | NA | NA |
| Age: 9 | 0.24 | 0.24 | NA |
| Age: 10 | 0.05 | 0.04* | 0.2 |
