# Supplementary material for: Reference values for psychoacoustic tests on Polish school children 7–10 years old
Source: PLoS One. 2019 Aug 28;14(8):e0221689. doi: 10.1371/journal.pone.0221689 (PMC6713444; doi:10.1371/journal.pone.0221689)
Supplement: S2 Table — (DOCX) [file pone.0221689.s003.docx]

**S2 Table**. **Results of various tests for normality to validate the underlying distribution of collected observations.** Asterisks (*) denote significance (p-value<0.05).

| Cramer–von Mises test | | | | | |
| --- | --- | --- | --- | --- | --- |
|  | Whole | Age: 7 | Age: 8 | Age: 9 | Age: 10 |
| CST | 0* | 0.38 | 0.05 | 0.07 | 0* |
| DDT_L | 0.28 | 0.93 | 0.44 | 0.87 | 0.78 |
| DDT_R | 0* | 0.04* | 0* | 0.09 | 0.34 |
| DPT | 0.39 | 0.75 | 0.39 | 0.48 | 0.93 |
| FPT | 0.05* | 0.02* | 0.56 | 0.76 | 0.16 |
| Anderson–Darling test | | | | | |
|  | Whole | Age: 7 | Age: 8 | Age: 9 | Age: 10 |
| CST | 0.00* | 0.43 | 0.06 | 0.07 | 0.00* |
| DDT_L | 0.28 | 0.88 | 0.37 | 0.85 | 0.74 |
| DDT_R | 0.00* | 0.04* | 0.00* | 0.11 | 0.35 |
| DPT | 0.39 | 0.75 | 0.27 | 0.46 | 0.84 |
| FPT | 0.05 | 0.03* | 0.71 | 0.75 | 0.13 |
| Lielliefors test | | | | | |
|  | Whole | Age: 7 | Age: 8 | Age: 9 | Age: 10 |
| CST | 0.00* | 0.37 | 0.08 | 0.01* | 0.00* |
| DDT_L | 0.28 | 0.92 | 0.64 | 0.87 | 0.87 |
| DDT_R | 0.00* | 0.21 | 0.00* | 0.12 | 0.10 |
| DPT | 0.39 | 0.66 | 0.18 | 0.74 | 0.95 |
| FPT | 0.05 | 0.07 | 0.63 | 0.59 | 0.25 |
| Wilk–Shapiro test | | | | | |
|  | Whole | Age: 7 | Age: 8 | Age: 9 | Age: 10 |
| CST | 0.00* | 0.35 | 0.11 | 0.06 | 0.02* |
| DDT_L | 0.28 | 0.74 | 0.49 | 0.87 | 0.75 |
| DDT_R | 0.00* | 0.06 | 0.00* | 0.21 | 0.29 |
| DPT | 0.39 | 0.72 | 0.14 | 0.33 | 0.56 |
| FPT | 0.05 | 0.06 | 0.86 | 0.59 | 0.08 |
