# Supplementary material for: Reference values for psychoacoustic tests on Polish school children 7–10 years old
Source: PLoS One. 2019 Aug 28;14(8):e0221689. doi: 10.1371/journal.pone.0221689 (PMC6713444; doi:10.1371/journal.pone.0221689)
Supplement: S3 Table — (DOCX) [file pone.0221689.s004.docx]

**S3 Table.** **Comparison of reference values calculated using different approaches.** P-value is calculated based on a two-sample Mann–Whitney nonparametric test. Asterisk (*) represents significance (p-value<0.05).

| DPT | | | | | | | |
| --- | --- | --- | --- | --- | --- | --- | --- |
| Age | N | Mean | SD | Median | Ref. Value  (10% quantile) | Ref. Value  (2SD) | *p*-value |
| 7-8 | 48 | 60.99 | 22.64 | 60 | 28.50 | 15.72 | 5e-04* |
| 9-10 | 46 | 75.43 | 15.39 | 75 | 53.75 | 44.66 | 5e-04* |
| FPT | | | | | | | |
| Age | N | Mean | SD | Median | Ref. Value  (10% quantile) | Ref. Value  (2SD) | *p*-value |
| 7-8 | 48 | 49.27 | 23.97 | 45.00 | 18.5 | 1.34 | 0.0362* |
| 9-10 | 46 | 59.78 | 23.96 | 61.25 | 27.5 | 11.86 | 0.0362* |
| CST | | | | | | | |
| Age | N | Mean | SD | Median | Ref. Value  (10% quantile) | Ref. Value  (2SD) | *p*-value |
| 7-8 | 48 | 80.67 | 9.85 | 82.85 | 68.55 | 60.96 | 4e-04* |
| 9-10 | 46 | 87.40 | 7.73 | 91.45 | 77.15 | 71.93 | 4e-04* |
| DDT Left | | | | | | | |
| Age | N | Mean | SD | Median | Ref. Value  (10% quantile) | Ref. Value  (2SD) | *p*-value |
| 7-8 | 48 | 55.73 | 17.22 | 57.5 | 34.25 | 21.29 | 2e-04* |
| 9-10 | 46 | 68.48 | 14.86 | 70.0 | 52.50 | 38.75 | 2e-04* |
| DDT Right | | | | | | | |
| Age | N | Mean | SD | Median | Ref. Value  (10% quantile) | Ref. Value  (2SD) | *p*-value |
| 7-8 | 48 | 76.93 | 14.98 | 82.5 | 56.0 | 46.98 | 0.0025* |
| 9-10 | 46 | 84.95 | 9.29 | 87.5 | 72.5 | 66.37 | 0.0025* |
