# Supplementary material for: Reference values for psychoacoustic tests on Polish school children 7–10 years old
Source: PLoS One. 2019 Aug 28;14(8):e0221689. doi: 10.1371/journal.pone.0221689 (PMC6713444; doi:10.1371/journal.pone.0221689)
Supplement: S4 Table — (DOCX) [file pone.0221689.s005.docx]

**S4 Table.** **Distribution of patients’ gender.**

| Gender | Age | | | |
| --- | --- | --- | --- | --- |
|  | 7 | 8 | 9 | 10 |
| Female | 15 | 22 | 10 | 9 |
| Male | 3 | 8 | 11 | 16 |
| Gender | Age | | | |
|  | 7-8 | | 9-10 | |
| Female | 37 | | 19 | |
| Male | 11 | | 27 | |
