# Supplementary material for: Reference values for psychoacoustic tests on Polish school children 7–10 years old
Source: PLoS One. 2019 Aug 28;14(8):e0221689. doi: 10.1371/journal.pone.0221689 (PMC6713444; doi:10.1371/journal.pone.0221689)
Supplement: S5 Table — (DOCX) [file pone.0221689.s006.docx]

**S5 Table.** **Exploration of age and gender effect.** Coefficients of linear regression model given by the equation: $score=\alpha+\beta_{1}\cdot age+\beta_{2}\cdot gender+\beta_{3}\cdot age\cdot gender$. Bold numbers are statistically significant coefficients.

| **Variable** | **DPT Test** | | **FPT Test** | | **CST Test** | | **DDT Test (Right Ear)** | | **DDT Test (Left Ear)** | |
| --- | --- | --- | --- | --- | --- | --- | --- | --- | --- | --- |
|  | coeff. | *p*-val | coeff. | *p*-val | coeff. | *p*-val | coeff. | *p*-val | coeff | *p*-val |
| Age ($\beta_{1}$) | **7.43** | **<0.01** | 0.46 | 0.88 | **3.50** | **<0.01** | **3.58** | **0.03** | **7.01** | **<0.01** |
| Gender ($\beta_{2}$) | -4.16 | 0.91 | -64.3 | 0.16 | 3.34 | 0.84 | -7.66 | 0.75 | 29.50 | 0.34 |
| Age:Gender ($\beta_{3}$) | 0.43 | 0.92 | 7.78 | 0.13 | -0.75 | 0.70 | 0.86 | 0.75 | -3.43 | 0.33 |
