# Supplementary material for: Reference values for psychoacoustic tests on Polish school children 7–10 years old
Source: PLoS One. 2019 Aug 28;14(8):e0221689. doi: 10.1371/journal.pone.0221689 (PMC6713444; doi:10.1371/journal.pone.0221689)
Supplement: S6 Table — (DOCX) [file pone.0221689.s007.docx]

**S6 Table. Influence of atypical observation.** Results of Kruskal-Wallis tests for significance of age with respect to the test scores

|  | p-value (Kruskal-Wallis) | |
| --- | --- | --- |
| Acoustic Test | Primary analysis | Analysis without observation id.65 |
| CST | 0.0061 | 0.0083 |
| DPT | 0.0021 | 0.0021 |
| FPT | 0.1433 | 0.1586 |
| DDT (left ear) | 0.0053 | 0.0070 |
| DDT (right ear) | 0.0200 | 0.0268 |
