# Supplementary material for: Reference values for psychoacoustic tests on Polish school children 7–10 years old
Source: PLoS One. 2019 Aug 28;14(8):e0221689. doi: 10.1371/journal.pone.0221689 (PMC6713444; doi:10.1371/journal.pone.0221689)

**S1 Fig.** **Distributions of test scores according to age group and gender.** They show a consistent increase in central auditory capabilities with age, but no effect with respect to gender. A) DPT Test. B) FPT Test. C) CST Test. D–F) DDT Test separated into scores for left (E) and right (F) ears and the difference in scores between right and left (D). Distributions are presented as standard boxplots with single points indicating outliers.


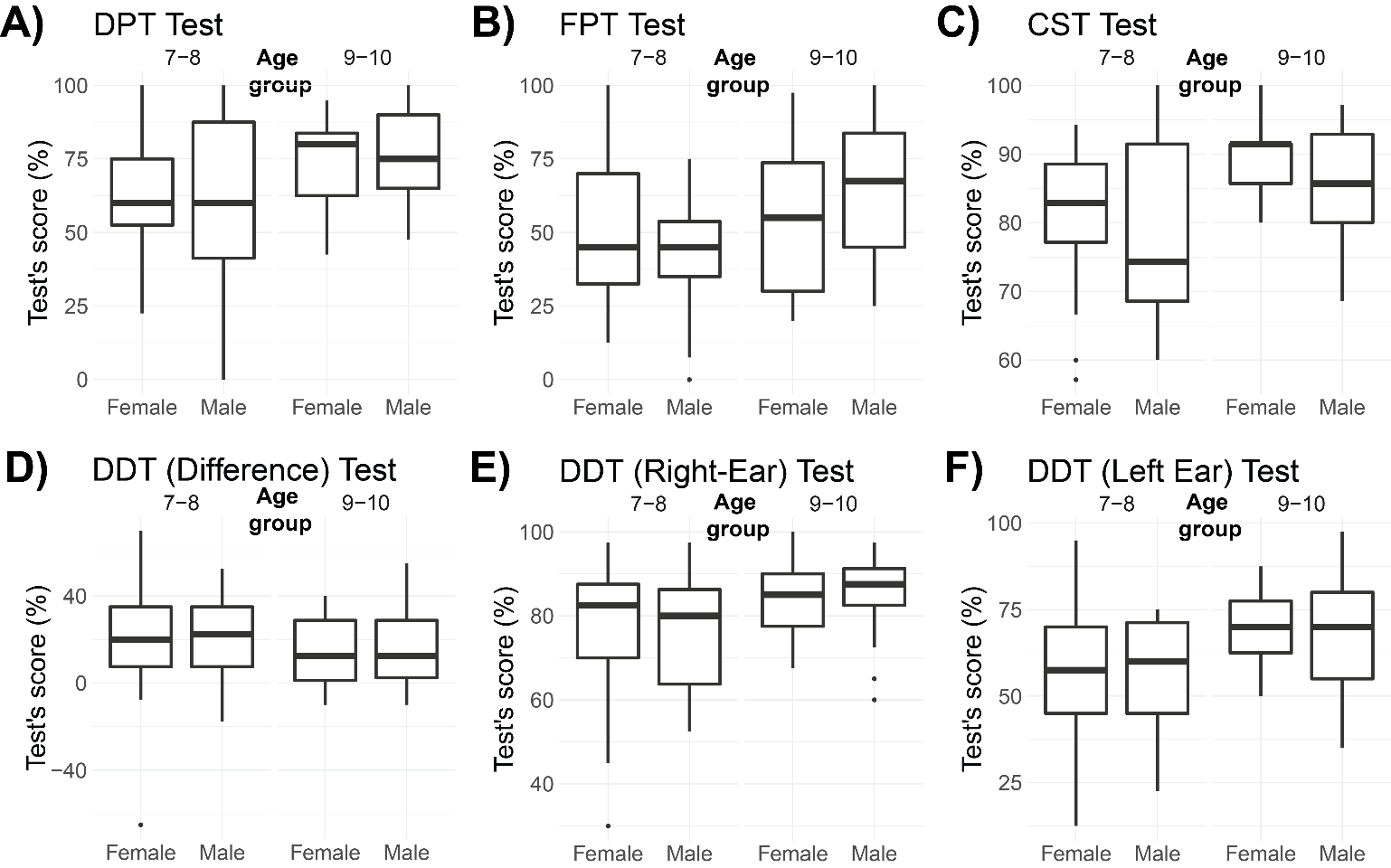

Supplement: S1 Fig — (DOCX) [file pone.0221689.s008.docx]
