# Supplementary material for: Reference values for psychoacoustic tests on Polish school children 7–10 years old
Source: PLoS One. 2019 Aug 28;14(8):e0221689. doi: 10.1371/journal.pone.0221689 (PMC6713444; doi:10.1371/journal.pone.0221689)

**S2 Fig.** **Correlation between test scores and Right Ear Advantage (REA).** REA calculated as the difference in DDT score between right and left ear. P-value of test for association between paired samples as implemented in cor.test from R base package, 0 indicates value below 0.005.


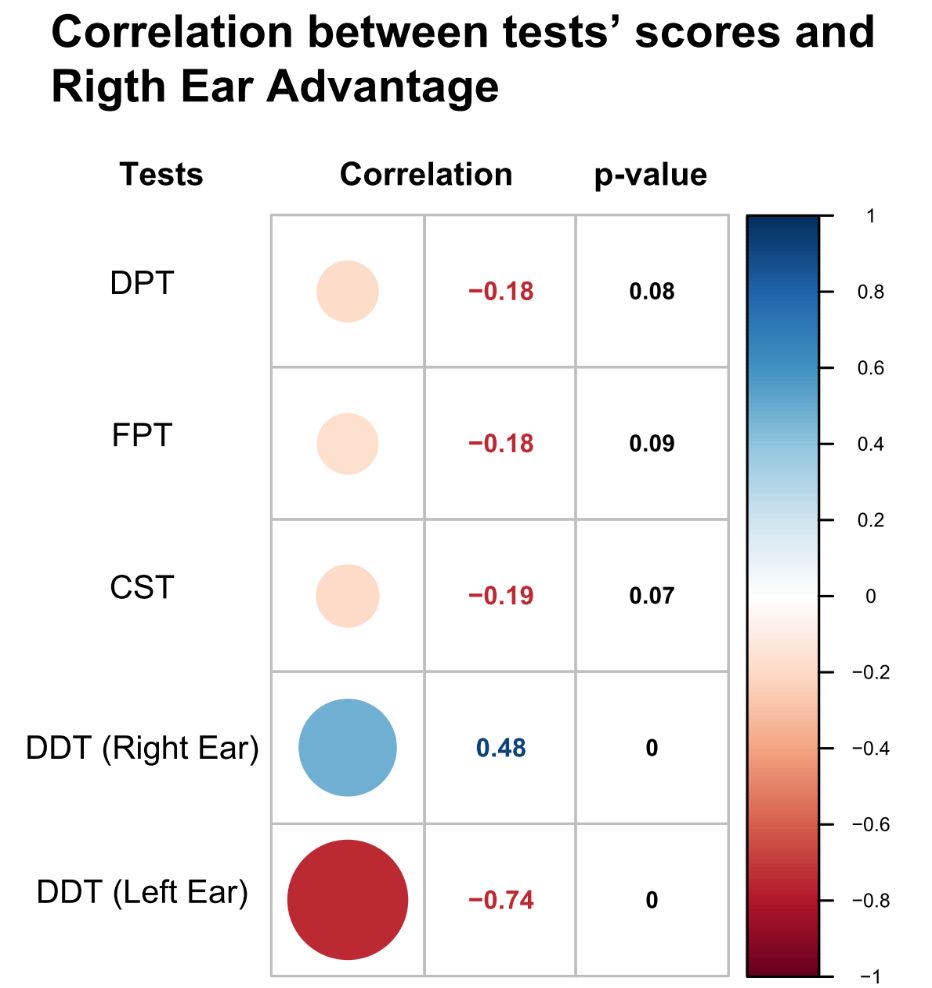

Supplement: S2 Fig — (DOCX) [file pone.0221689.s009.docx]
